# A cross-sectional analysis of ITN and IRS coverage in Namibia in 2013

**DOI:** 10.1186/s12936-018-2417-z

**Published:** 2018-07-16

**Authors:** Sophie H. Allcock, Elizabeth H. Young, Manjinder S. Sandhu

**Affiliations:** 1 Department of Medicine, University of Cambridge, Cambridge, Cambridgeshire UK; 2 Wellcome Sanger Institute, Hinxton, Cambridgeshire CB10 1SA UK

**Keywords:** Malaria, Indoor residual spraying, Insecticide-treated nets, Vector control, Namibia

## Abstract

**Background:**

Achieving vector control targets is a key step towards malaria elimination. Because of variations in reporting of progress towards vector control targets in 2013, the coverage of these vector control interventions in Namibia was assessed.

**Methods:**

Data on 9846 households, representing 41,314 people, collected in the 2013 nationally-representative Namibia Demographic and Health Survey were used to explore the coverage of two vector control methods: indoor residual spraying (IRS) and insecticide-treated nets (ITNs). Regional data on *Plasmodium falciparum* parasite rate in those aged 2–10 years (*Pf*PR_2–10_), obtained from the Malaria Atlas Project, were used to provide information on malaria transmission intensity. Poisson regression analyses were carried out exploring the relationship between household interventions and *Pf*PR_2–10_, with fully adjusted models adjusting for wealth and residence type and accounting for regional and enumeration area clustering. Additionally, the coverage as a function of government intervention zones was explored and models were compared using log-likelihood ratio tests.

**Results:**

Intervention coverage was greatest in the highest transmission areas (*Pf*PR_2–10_ ≥ 5%), but was still below target levels of 95% coverage in these regions, with 27.6% of households covered by IRS, 32.3% with an ITN and 49.0% with at least one intervention (ITN and/or IRS). In fully adjusted models, *Pf*PR_2–10_ ≥ 5% was strongly associated with IRS (RR 14.54; 95% CI 5.56–38.02; p < 0.001), ITN ownership (RR 5.70; 95% CI 2.84–11.45; p < 0.001) and ITN and/or IRS coverage (RR 5.32; 95% CI 3.09–9.16; p < 0.001).

**Conclusions:**

The prevalence of IRS and ITN interventions in 2013 did not reflect the Namibian government intervention targets. As such, there is a need to include quantitative monitoring of such interventions to reliably inform intervention strategies for malaria elimination in Namibia.

**Electronic supplementary material:**

The online version of this article (10.1186/s12936-018-2417-z) contains supplementary material, which is available to authorized users.

## Background

Malaria is a global public health concern, causing approximately 438,000 deaths, worldwide, in 2015 [1]. The World Health Organization (WHO) Africa Region experiences a disproportionately high burden of malaria, with 88% of global cases in 2015 occurring in the region [1]. Namibia is one of eight sub-Saharan African countries aiming to eliminate malaria, and intends to eliminate by 2020.

Interventions for malaria control and elimination include indoor residual spraying (IRS), insecticide-treated nets (ITNs) and long-lasting insecticide-treated nets (LLINs). These are effective tools for reducing the adult mosquito population density and longevity, and are therefore fundamental for interrupting transmission [2]. ITNs and LLINs have successfully reduced the risk of infection in a number of settings [3–5], with up to 90% reductions in malaria transmission recorded following ITN implementation in some high-transmission settings [6]. High coverage of ITNs and IRS can both result in community-level protection [7, 8], highlighting the importance of high coverage and uptake of these interventions. There is also evidence to suggest that using IRS and ITNs in combination is more effective at reducing the vector population and interrupting transmission than ITNs alone [9, 10].

Malaria transmission in Namibia is heterogeneous. In 2013, it was estimated that 67% of Namibia’s population were living in the highest transmission areas [11]. Prevalence of malaria is highest in the northern regions that border Angola [12]. Namibia has experienced fluctuations in malaria incidence with reported cases rising from 4911 in 2013 [11] to 15,915 in 2014 [1], with two outbreaks occurring in 2016 and 2017 [13–16]. Importantly, between 2000 and 2015, Namibia’s overall malaria incidence and mortality rates increased by over 20% [17], highlighting the need for an effective elimination programme.

Namibia’s 2010–2016 Malaria Strategic Plan (MSP) aimed to achieve at least 95% coverage with a combination of vector control interventions in all malaria endemic areas and identified transmission foci by 2013 [12]. However, the 2013 Namibia Demographic and Health Survey (DHS), a nationally-representative survey that collected data on IRS and ITN coverage, reported that only 24% of households had at least one ITN, and just 16% of households had received IRS during the previous 12 months [18]. By contrast, a governmental report indicated that IRS was successfully completed in the eight malaria regions, with 93% coverage of targeted households achieved by the end of January 2013 [19]. To understand these discordant findings, a detailed analysis of ITN and IRS coverage was conducted as a function of DHS data, malaria transmission patterns and government intervention zones across Namibia in 2013.

## Methods

### Ethical considerations

Data used in these analyses were available through the DHS Programme [20]. Ethical review and approval for procedures and questionnaires for standard DHS surveys is provided by the ICF Institutional Review Board (IRB). Country-specific DHS survey protocols are reviewed by the ICF IRB and typically by an IRB in the host country. Verbal consent is obtained from the participant and a signature is provided by the interviewer to acknowledge that this event has taken place. Displaced geographical coordinates were obtained following approval from the DHS Programme. Data were securely stored separately from individual and household data.

### Data sources

The DHS programme conducts standardized, nationally-representative surveys in over 90 countries worldwide, collecting data pertaining to the broad themes of fertility, family planning, maternal and child health, human immunodeficiency virus (HIV), malaria, and nutrition [21]. The methods of the 2013 Namibia DHS are detailed elsewhere [18]. In summary, the survey used a two-stage stratified cluster design, which involved dividing each administrative region into enumeration areas (EAs) and then classifying these EAs as either urban or rural. EAs were then selected from the urban and rural strata and around 20 households per EA were selected for the survey [18]. The DHS involved three surveys: the Household survey, the Woman’s survey and the Man’s survey [18]. The household wealth index was calculated using principal component analysis involving economic indicators such as household assets [22, 23].

Available data on vector control indicators, collected as part of the DHS Household survey, included data pertaining to ITNs and IRS. A household member was asked to show all the mosquito nets to the interviewer and identify which household members slept under each net the night before the survey. IRS coverage was determined by asking a household member if the dwelling had been sprayed against mosquitoes in the last 12 months. DHS definitions of IRS and ITN were as follows:

- *Indoor residual spraying* Spraying of the interior walls of the dwelling with an insecticide against mosquitoes.
- *Insecticide-treated net* A factory-treated net that does not require any further treatment (LLIN), or a pre-treated net obtained in the past 12 months, or a net that has been soaked with insecticide within the past 12 months.

Households were classified as not having an ITN if the household did not have any mosquito net or only had untreated nets. Households with at least one ITN per two people who slept in the household the night before the survey were classified as having a sufficient number of ITNs.

EA coordinates were obtained from the DHS Programme. EA coordinates represent a group of up to 20 households and are randomly displaced. Rural EAs are randomly displaced by up to 5 km and urban EAs are displaced by up to 2 km [24].

The indicator *Plasmodium falciparum* parasite rate (*Pf*PR) is a commonly used indicator of malaria transmission intensity. *Pf*PR_2–10_ is the proportion of the population aged 2–10 years carrying asexual blood parasites [25]. Modelled malaria parasite prevalence data for the year 2013 were obtained from the Malaria Atlas Project (MAP) portal, made available under the Creative Commons Attribution 3.0 Unported License [26, 27]. MAP *Pf*PR_2–10_ estimates were derived from data collected across 27,573 population clusters from 1995 to 2014, which were adjusted for age, season and the diagnostic test used [28]. This model was used to predict *Pf*PR_2–10_ for malaria-endemic countries across Africa, including Namibia, from the year 2000 to 2015, at a resolution of 5 × 5 km [28].

Malaria zones were assigned in line with MSP district strata outlined in the MSP documentation [12]. As part of Namibia’s 2010–2016 MSP, the objective for integrated vector control was to achieve at least 95% coverage with a combination of vector control interventions in all malaria endemic areas and identified transmission foci by 2013 [12]. The country was divided into three Zones, with Zone 1 representing the highest transmission areas (moderate transmission risk), Zone 2 representing low transmission risk and Zone 3 for “risk free” areas [12]. Vector control targets were set for each zone. In Zone 1 the aim was to achieve 95% coverage of a combination of IRS and ITNs in addition to winter larviciding [12]. In Zone 2 IRS, ITNs and larviciding were to be targeted to selected foci [12].

For spatial representations of data, shapefiles for Namibia were downloaded from DIVA-GIS [29], originally sourced from the Database of Global Administrative Areas (GADM) [30].

### Data analysis and statistical methods

Quantum GIS (QGIS) 2.14.1 was used for all maps and spatial analyses. All statistical analyses were carried out using STATA 14.0 software package (StataCorp: College Station, TX, USA). All households captured in the survey period (May to September 2013) were included in the subsequent analyses, giving a total of 9846 households and a population of 41,314 individuals.

Three models of transmission intensity were constructed. The first classified households according to weighted regional *Pf*PR_2–10_ values obtained from MAP for the year 2013. Regions were classified into three categories based on their *Pf*PR_2–10_ values. The < 1% category constitutes very low transmission risk or malaria-free areas, the 1 to < 5 % category represents low transmission risk and the ≥ 5% category signifies moderate risk of transmission. Regions with *Pf*PR_2–10_ estimates of zero (malaria-free) were classified into the < 1% category.

The second model used raster data for *Pf*PR_2–10_ obtained from MAP for the year 2013. *Pf*PR_2–10_ values for each EA were assigned using the “Point Sampling Tool” in QGIS 2.14.1 [31]. Raster values were converted to percentages and were similarly classified into three *Pf*PR_2–10_ categories: < 1; 1 to < 5 and ≥ 5 %. Where no raster values were available for EAs because they were located in areas where no transmission was predicted to occur, the EAs were assigned the value of zero. To account for random displacement in DHS data, Euclidean buffers were drawn around EA points of 2 km for urban EAs and 5 km for rural EAs. The MAP *Pf*PR_2–10_ raster surface was overlaid with buffered EA locations and the mean *Pf*PR_2–10_ value was extracted. A high correlation between extracted mean *Pf*PR_2–10_ values and extracted point *Pf*PR_2–10_ values was observed. EAs were re-categorized into *Pf*PR_2–10_ categories (< 1, 1 to < 5, > 5%) according to the mean *Pf*PR_2–10_ values.

In additional sensitivity analyses, EAs outside of the boundary of the *Pf*PR_2–10_ raster were assigned the value of the nearest raster cell up to 5 km away, relative to the maximum EA displacement distance. This was repeated to assign EAs up to 10 and 20 km outside of the raster boundary the value of the nearest cell. EAs were re-categorized into *Pf*PR_2–10_ categories (< 1, 1 to < 5, > 5%) and explored the coverage of IRS, having an ITN and having either intervention for the three models respectively (assigning raster cell values to EAs up to 5, 10 and 20 km away).

The third model classified households according to MSP zones. Zones were assigned using QGIS 2.14.1. Administrative districts were assigned zones 1, 2 or 3, as defined by the MSP, and EAs were mapped. To assign zones to EAs, polygon attributes were assigned to the EA points using the QGIS 2.14.1 “Join Attributes by Location” tool.

Categorical data are presented as a frequency and percentage. p-values were calculated using a Chi squared test and p < 0.05 was considered statistically significant. Primary analyses were unweighted but additional weighted analyses were carried out to make the data representative of the whole population. Weighted analyses used the DHS weight variable as per DHS Programme guidance [32]. First a univariable Poisson model (STATA ‘poisson’ function) was used to test for the association between IRS and regional *Pf*PR_2–10_. In the second model, EA and region were added as mixed effects (STATA ‘mepoisson’ function). In the third model, wealth and residence type covariates were additionally adjusted for. These analyses were carried out for the other outcomes of interest: whether a household owned at least one ITN, and whether a household had at least one intervention (ITN and/or IRS). Risk ratios are presented with 95% confidence intervals and the p-value.

Log-likelihood ratio tests were carried out to compare regional *Pf*PR_2–10_, EA *Pf*PR_2–10_ and MSP zones. The first model tested the association between regional *Pf*PR_2–10_ and IRS, adjusted for covariates (wealth and residence type) and accounted for regional and EA clustering. The second model additionally adjusted for EA *Pf*PR_2–10_. The third model adjusted for MSP zones in addition to model 1. Log-likelihood ratio tests were carried out with models 2 and 3, respectively nested in model 1.

Log-likelihood ratio tests were repeated for the additional models of EA *Pf*PR_2–10_. The mean EA *Pf*PR_2–10_ model was compared to the regional *Pf*PR_2–10_ model for each intervention using log-likelihood ratio tests. First, the association between regional *Pf*PR_2–10_ and IRS was tested, adjusting for regional and EA clustering, wealth and residence type. The second model additionally adjusted for the mean EA *Pf*PR_2–10_ and a log-likelihood ratio test was conducted with the second model nested in the first. This was repeated for the association with having an ITN and either intervention.

Further, EA *Pf*PR_2–10_ models, where EAs were assigned raster cell values at up to 5, 10 and 20 km away, were compared to the regional *Pf*PR_2–10_ model, respectively, for each intervention (IRS, ITN and either intervention). First, the association between regional *Pf*PR_2–10_ and having IRS was tested, adjusting for regional and EA clustering, wealth and residence type. The second model additionally adjusted for EA *Pf*PR_2–10_ and a log-likelihood ratio test was carried out with the second model nested in the first. This was repeated for each model of EA *Pf*PR_2–10_ and for each intervention.

## Results

### Study characteristics

Analyses included 9846 households representing 41,314 individuals. Households were distributed across 550 EAs. There were a total of 4763 urban and 5083 rural households, and 50.2% of households were in the highest transmission areas (*Pf*PR_2–10_ ≥ 5%) (Table 1).

**Table 1:** Background characteristics of households surveyed

Background characteristics	Distribution of households No. (%)
Residence type	
Urban	4763 (48.4)
Rural	5083 (51.6)
Wealth quintile	
Lowest	1696 (17.2)
Second	1945 (19.8)
Middle	2012 (20.4)
Fourth	2178 (22.1)
Highest	2015 (20.5)
Regional *Pf*PR_2–10_ (%)	
< 1	3467 (35.2)
1 to < 5	1432 (14.5)
> 5	4947 (50.2)
EA *Pf*PR_2–10_ (%)	
< 1	4184 (42.5)
1 to < 5	1082 (11.0)
> 5	4580 (46.5)
MSP zone	
3	3588 (36.4)
2	2.033 (20.7)
1	4225 (42.9)
IRS coverage^a^	
No IRS	7921 (80.5)
IRS	1676 (17.0)
Don’t know	245 (2.5)
ITN coverage	
No net	6533 (66.4)
Untreated net	940 (9.6)
ITN	2373 (24.1)
Number of ITNs in household	
0	7473 (75.9)
1	1142 (11.6)
> 1	1231 (12.5)
ITN per two people	
< 1 ITN per two people	8724 (88.6)
≥ 1 ITN per two people	1122 (11.4)
Total	9846 (100.0)

*Pf*PR_2–10_, *Plasmodium falciparum* parasite rate in those aged 2–10 years; EA, enumeration area; MSP, Malaria Strategic Plan; IRS, indoor residual spraying; ITN, insecticide-treated net

^a^n = 9842

**Fig. 1:**
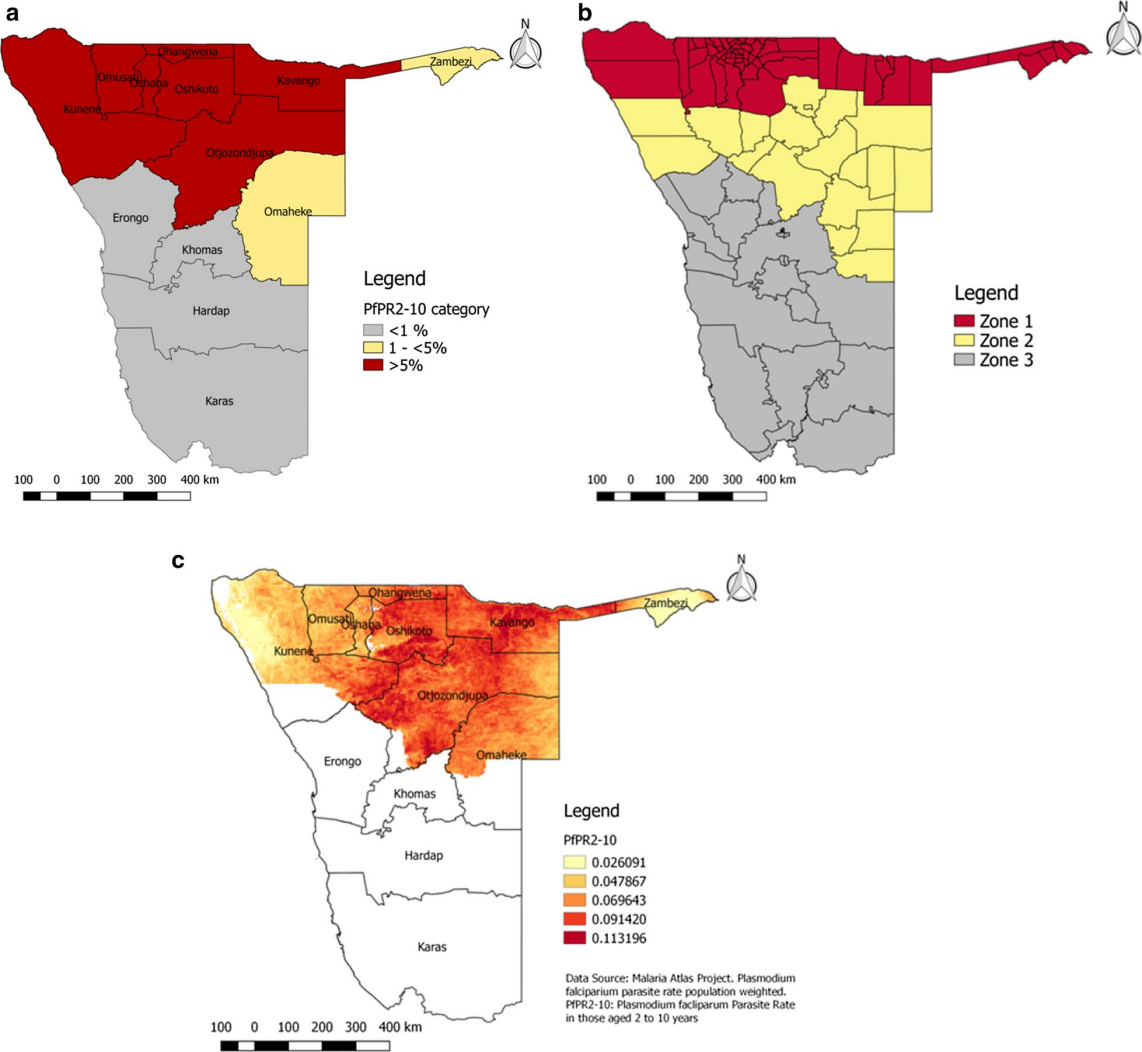
Three models of transmission intensity in Namibia. **a** Regional *Pf*PR_2–10_ used to classify regions into three categories; **b** Namibia classified according to Malaria Strategic Plan (MSP) defined zones; **c** *Pf*PR_2–10_ values used to classify enumeration areas (EAs) into three categories. All *Pf*PR_2–10_ data sourced from the Malaria Atlas Project [26]. *Pf*PR_2–10_, *Plasmodium falciparum* parasite rate in those aged 2–10 years (Data source: Malaria Atlas Project)

Malaria transmission intensity was highest among the northern and north-eastern regions of Namibia in 2013 across all three models (Fig. 1a–c). The highest transmission regions (*Pf*PR_2–10_ ≥ 5%) were Kunene, Omusati, Oshana, Oshikoto, Ohangwena, Otjozondjupa and Kavango (Fig. 1a). Low transmission occurred in Zambezi and Omaheke (*Pf*PR_2–10_ 1 to < 5%).

The results describe unweighted household-level ITN and IRS coverage. In secondary weighted analyses, there was no material difference in prevalence estimates observed for intervention coverage. ITN and IRS coverage were primarily explored as a function of regional *Pf*PR_2–10_.

### Household IRS coverage

**Fig. 2:**
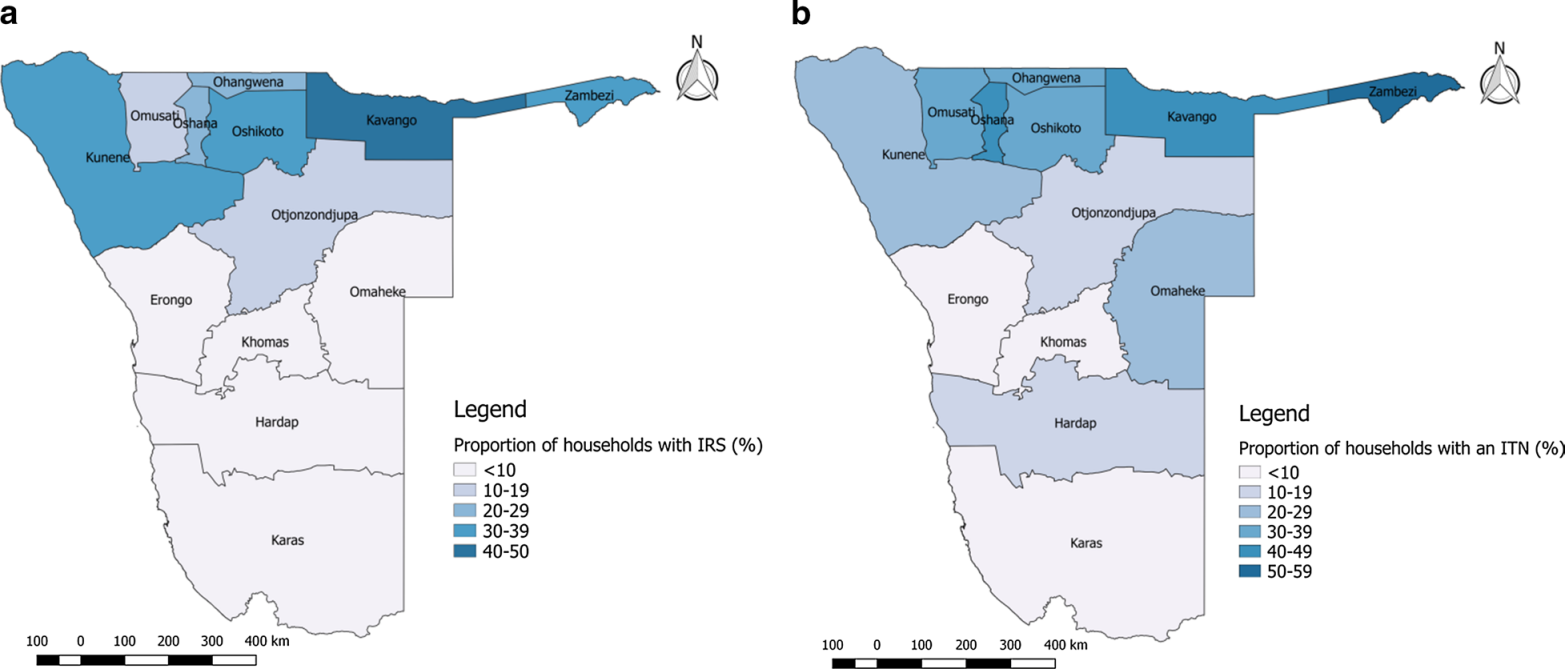
Regional household coverage of ITNs and IRS. **a** Proportion of households in each region that reported receiving IRS in the previous 12 months; **b** proportion of households in each region with at least one ITN. ITN, insecticide-treated net; IRS, indoor residual spraying

Only 17.0% of households were sprayed in Namibia in 2013 (Table 1). Of these households, 91.0% reported that the dwelling was sprayed by the government. A higher proportion of rural households received IRS compared with urban households (28.8% vs 4.5%) (Table 2). The highest proportion of households sprayed were in the Kavango region (Fig. 2a) and overall IRS coverage was highest in the northern regions, in line with the geographical distribution of malaria transmission intensity (Fig. 1a). Similarly, IRS coverage was highest in the ≥ 5% *Pf*PR_2–10_ category at 27.6% and was 18.9% in the 1 to < 5% category, again suggesting that IRS was targeted to higher transmission areas.

**Table 2:** IRS and ITN coverage by household background characteristics

Household characteristics	IRS in previous 12 months			p-value	ITN ownership		p-value	ITN and/or IRS		p-value	ITN and IRS		p-value
	No	Yes	Don’t know		No ITN	At least one ITN		None	ITN and/or IRS		One or no interventions	Both ITN and IRS	
	No. (%)	No. (%)	No. (%)		No. (%)	No. (%)		No. (%)	No. (%)		No. (%)	No. (%)	
Residence type													
Urban	4433 (93.1)	214 (4.5)	113 (2.4)	< 0.001	4017 (84.3)	746 (15.7)	< 0.001	3782 (81.4)	865 (18.6)	< 0.001	4566 (98.3)	81 (1.7)	< 0.001
Rural	3488 (68.6)	1462 (28.8)	132 (2.6)		3456 (68.0)	1627 (32.0)		2588 (52.3)	2362 (47.7)		4257 (86.0)	693 (14.0)	
Wealth quintile													
Lowest	1107 (65.3)	561 (33.1)	28 (1.7)	< 0.001	1145 (67.5)	551 (32.5)	< 0.001	808 (48.4)	860 (51.6)	< 0.001	1426 (85.5)	242 (14.5)	< 0.001
Second	1440 (74.1)	452 (23.3)	52 (2.7)		1388 (71.4)	557 (28.6)		1114 (58.9)	778 (41.1)		1668 (88.2)	224 (11.8)	
Middle	1634 (81.2)	327 (16.3)	51 (2.5)		1470 (73.1)	542 (26.9)		1265 (64.5)	696 (35.5)		1797 (91.6)	164 (8.4)	
Fourth	1873 (86.1)	227 (10.4)	76 (3.5)		1727 (79.3)	451 (20.7)		1544 (73.5)	556 (26.5)		1993 (94.9)	107 (5.1)	
Highest	1867 (92.7)	109 (5.4)	38 (1.9)		1743 (86.5)	272 (13.5)		1639 (83.0)	337 (17.1)		1939 (98.1)	37 (1.9)	
Regional *Pf*PR_2–10_ (%)													
< 1	3341 (96.4)	41 (1.2)	82 (2.4)	< 0.001	3254 (93.9)	213 (6.1)	< 0.001	3141 (92.9)	241 (7.1)	< 0.001	3374 (99.8)	8 (0.2)	< 0.001
1 to < 5	1137 (79.4)	270 (18.9)	25 (1.8)		870 (60.8)	562 (39.3)		778 (55.3)	629 (44.7)		1213 (86.2)	194 (13.8)	
> 5	3443 (69.6)	1365 (27.6)	138 (2.8)		3349 (67.7)	1598 (32.3)		2451 (51.0)	2357 (49.0)		4236 (88.1)	572 (11.9)	
EA *Pf*PR_2–10_ (%)													
< 1	3959 (94.7)	122 (2.9)	100 (2.4)	< 0.001	3811 (91.1)	373 (8.9)	< 0.001	3639 (89.2)	442 (10.8)	< 0.001	4039 (99.0)	42 (1.0)	< 0.001
1 to < 5	721 (66.6)	327 (30.2)	34 (3.1)		560 (51.8)	522 (48.2)		422 (40.3)	626 (59.7)		837 (79.9)	211 (20.1)	
> 5	3241 (70.8)	1227 (26.8)	111 (2.4)		3102 (67.7)	1478 (32.3)		2309 (51.7)	2159 (48.3)		3947 (88.3)	521 (11.7	
MSP zones													
3	3459 (96.5)	43 (1.2)	83 (2.3)	< 0.001	3363 (93.7)	225 (6.3)	< 0.001	3249 (92.8)	253 (7.2)	< 0.001	3493 (99.7)	9 (0.3)	< 0.001
2	1703 (83.8)	282 (13.9)	48 (2.4)		1629 (80.1)	404 (19.9)		1392 (70.1)	593 (29.9)		1869 (95.5)	89 (4.5)	
1	2759 (65.3)	1351 (32.0)	114 (2.7)		2481 (58.7)	1744 (41.3)		1729 (42.1)	2381 (57.9)		3434 (83.6)	676 (16.5)	
Total	7921 (80.5)	1676 (17.0)	245 (2.5)		7473 (75.9)	2373 (24.1)		6370 (66.4)	3227 (33.6)		8823 (91.9)	774 (8.1)	

IRS, indoor residual spraying; ITN, insecticide-treated net; *Pf*PR_2–10_, *Plasmodium falciparum* parasite rate in ages 2–10 years; EA, enumeration area; MSP, Malaria Strategic Plan

p-value corresponds to Chi squared test

In multivariable mixed effects analyses, regional *Pf*PR_2–10_ was significantly positively associated with IRS, with households in the ≥ 5% category most likely to have been sprayed (RR 14.54; 95% CI 5.56–38.02) (Table 3). Rural residence type was also strongly significantly associated with IRS coverage (RR 5.02; 95% CI 3.83–6.58). Some evidence was found for a modest and positive association between wealth and IRS coverage (Table 3). However, sensitivity analyses indicated that this relationship was inconsistent across urban and rural areas (Additional file 1: Table S1).

**Table 3:** Multivariable association between IRS and exposures of interest, adjusted for regional, enumeration area and household clustering in Namibia 2013 (n = 9597)

Exposures of interest	Model 1		Model 2		Model 3	
	RR (95% CI)	p-value	RR (95% CI)	p-value	RR (95% CI)	p-value
Residence type						
Urban	1.00 (reference)		1.00 (reference)		1.00 (reference)	
Rural	6.41 (5.56–7.40)	< 0.001	4.53 (3.5–5.9)	< 0.001	5.02 (3.83–6.58)	< 0.001
Wealth quintile						
Lowest	1.00 (reference)		1.00 (reference)		1.00 (reference)	
Second	0.71 (0.63–0.80)	< 0.001	1.12 (0.98–1.28)	0.097	1.16 (1.02–1.33)	0.029
Middle	0.50 (0.43–0.57)	< 0.001	1.11 (0.95–1.30)	0.189	1.20 (1.03–1.40)	0.023
Fourth	0.32 (0.28–0.37)	< 0.001	1.02 (0.84–1.25)	0.813	1.25 (1.03–1.51)	0.021
Highest	0.16 (0.13–0.20)	< 0.001	1.11 (0.84–1.47)	0.449	1.63 (1.25–2.13)	< 0.001
Regional *Pf*PR_2–10_ (%)						
< 1	1.00 (reference)		1.00 (reference)		1.00 (reference)	
1 to < 5	15.83 (11.40–22.0)	< 0.001	11.00 (3.18–38.07)	< 0.001	5.82 (1.60–21.22)	0.008
> 5	23.42 (17.16–31.95)	< 0.001	27.12 (10.76–68.35)	< 0.001	14.54 (5.56–38.02)	< 0.001

Model 1, univariable association between exposures of interest and IRS coverage; Model 2, adjusted for regional and enumeration area clustering; Model 3, additionally adjusted for all other exposures of interest in the table; *Pf*PR_2–10_, *Plasmodium falciparum* parasite rate in those aged 2–10 years; IRS, indoor residual spraying

### Household ITN ownership

Overall, 66.4% of households did not own a net of any kind, 9.6% owned only an untreated net and 24.1% owned at least one ITN (Table 1). Only 11.4% of all households had sufficient ITNs for at least one ITN per two people (Table 1).

ITN ownership was highest in the *Pf*PR_2–10_ 1 to < 5% category, with 39.3% of households owning an ITN, followed by 32.3% in the *Pf*PR_2–10_ ≥ 5% category (Table 2). A higher proportion of rural households owned an ITN compared with urban households (32.0% vs 15.7%) (Table 2). As expected, there was geographical heterogeneity in ITN ownership. A higher proportion of households in the northern and north-eastern regions owned an ITN, with Zambezi having the highest proportion of households owning at least one ITN (> 50%) (Fig. 2b).

In multivariable mixed effects analyses, households in the *Pf*PR_2–10_ 1 to < 5% category were most likely to own an ITN (RR 5.92; 95% CI 2.83–12.38) (Table 4). In these analyses, rural households were significantly more likely to own an ITN than urban households (RR 1.32; 95% CI 1.15–1.51). Again, there was some evidence to suggest a modest and positive association between wealth and ITN ownership (Table 4). However, this was not consistent across urban and rural residence types (Additional file 1: Table S2).

**Table 4:** Multivariable association between ITN ownership and exposures of interest, accounting for regional, enumeration area and household clustering in Namibia 2013 (n = 9842)

Exposures of interest	Model 1		Model 2		Model 3	
	Risk ratio (95% CI)	p-value	Risk ratio (95% CI)	p-value	Risk ratio (95% CI)	p-value
Residence						
Urban	1.00 (reference)		1.00 (reference)		1.00 (reference)	
Rural	2.04 (1.87–2.23)	< 0.001	1.17 (1.03–1.32)	0.017	1.32 (1.15–1.51)	< 0.001
Wealth quintile						
Lowest	1.00 (reference)		1.00 (reference)		1.00 (reference)	
Second	0.88 (0.78–0.99)	0.036	1.17 (1.03–1.32)	0.014	1.20 (1.06–1.36)	0.004
Middle	0.83 (0.74–0.93)	0.002	1.32 (1.16–1.51)	< 0.001	1.39 (1.21–1.58)	< 0.001
Fourth	0.64 (0.56–0.72)	< 0.001	1.36 (1.17–1.57)	< 0.001	1.48 (1.27–1.72)	< 0.001
Highest	0.42 (0.36–0.48)	< 0.001	1.29 (1.08–1.54)	0.005	1.49 (1.23–1.80)	< 0.001
Regional *Pf*PR_2–10_ (%)						
< 1	1.00 (reference)		1.00 (reference)		1.00 (reference)	
1 to < 5	6.39 (5.46–7.48)	< 0.001	5.96 (2.94–12.12)	< 0.001	5.92 (2.83–12.38)	< 0.001
> 5	5.26 (4.56–6.07)	< 0.001	5.36 (3.19–9.02)	< 0.001	5.32 (3.09–9.16)	< 0.001

Model 1, univariable association between exposures of interest and ITN coverage; Model 2, adjusted for regional and enumeration area clustering; Model 3, additionally adjusted for all other exposures of interest in the table; *Pf*PR_2–10_, *Plasmodium falciparum* parasite rate in those aged 2–10 years; ITN, insecticide-treated net

### ITN or IRS coverage

**Fig. 3:**
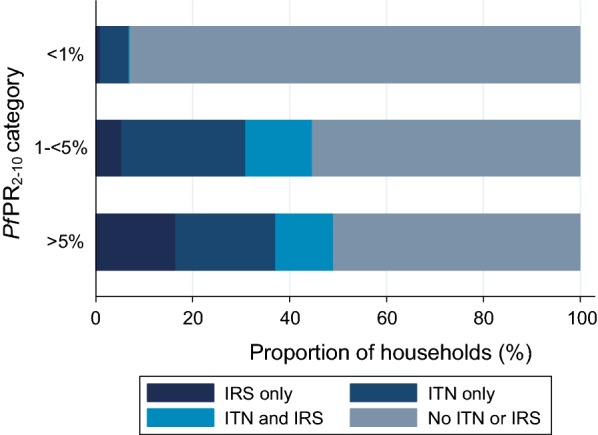
ITN and IRS coverage by *Pf*PR_2–10_ category in Namibia 2013. The proportion of households in each *Pf*PR_2–10_ category that had only IRS, only an ITN, an ITN and IRS and neither an ITN or IRS. ITN, insecticide-treated net; IRS indoor residual spraying; *Pf*PR_2–10_, *Plasmodium falciparum* parasite rate in those aged 2–10 years

Across the country, 33.6% of households had at least one intervention (ITN and/or IRS) (Table 2). In the highest transmission areas, 49.0% of households had at least one intervention (Table 2). In the highest transmission areas (*Pf*PR_2–10_ ≥ 5%), 51% of households had neither an ITN or IRS, 16.5% had IRS only, 20.6% had only an ITN and 11.9% had both an ITN and IRS (Fig. 3). Households in rural areas were more likely to have at least one intervention (47.7% vs 18.6%) and a higher proportion of rural households had both interventions (14.0% vs 1.7%) (Table 2).

Transmission intensity was strongly associated with intervention coverage, with households in the *Pf*PR_2–10_ ≥ 5% category the most likely to have at least one intervention (RR 6.10; 95% CI 3.74–9.97; p < 0.001)(Table 5). This suggests a targeting of these interventions to the higher transmission areas. Rural residence type was also associated with a significantly higher coverage with at least one intervention (RR 1.62; 95% CI 1.45–1.81). A significant positive association between wealth and coverage with at least one intervention was observed (Table 5).

**Table 5:** Multivariable association of exposures of interest with coverage of IRS and/or an ITN in Namibia in 2013, accounting for clustering and adjusting for covariates (n = 9597)

Exposure of interest	Model 1		Model 2		Model 3	
	Risk ratio (95% CI)	p-value	Risk ratio (95% CI)	p-value	Risk ratio (95% CI)	p-value
Residence type						
Urban	1.00 (reference)		1.00 (reference)		1.00 (reference)	
Rural	2.56 (2.37–2.77)	< 0.001	1.46 (1.33–1.63)	< 0.001	1.62 (1.45–1.81)	< 0.001
Wealth quintile						
Lowest	1.00 (reference)		1.00 (reference)		1.00 (reference)	
Second	0.80 (0.72–0.88)	< 0.001	1.05 (0.95–1.16)	0.370	1.12 (1.01–1.24)	0.032
Middle	0.69 (0.62–0.76)	< 0.001	1.09 (0.97–1.21)	0.139	1.22 (1.10–1.37)	< 0.001
Fourth	0.51 (0.46–0.57)	< 0.001	1.05 (0.93–1.18)	0.474	1.29 (1.14–1.46)	< 0.001
Highest	0.33 (0.29–0.38)	< 0.001	0.99 (0.86–1.15)	0.929	1.36 (1.16–1.60)	< 0.001
Regional *Pf*PR_2–10_ (%)						
< 1	1.00 (reference)		1.00 (reference)		1.00 (reference)	
1 to < 5	6.27 (5.41–7.28)	< 0.001	5.72 (2.94–11.11)	< 0.001	5.05 (2.59–9.85)	< 0.001
> 5	6.88 (6.03–7.85)	< 0.001	6.96 (4.28–11.31)	< 0.001	6.10 (3.74–9.97)	< 0.001

Model 1, univariable association between exposures of interest and IRS and/or ITN coverage; Model 2, adjusted for regional and enumeration area clustering; Model 3, additionally adjusted for all other exposures of interest in the table; *Pf*PR_2–10_, *Plasmodium falciparum* parasite rate in those aged 2–10 years; ITN, insecticide-treated net; IRS, indoor residual spraying

### Patterns of transmission intensities and ITN and IRS coverage

**Fig. 4:**
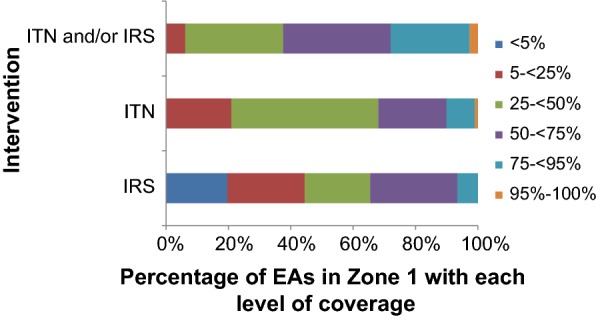
The percentage of enumeration areas in MSP Zone 1 that achieved each level of coverage for each intervention. *EA* enumeration area, *MSP* Malaria Strategic Plan, *IRS* indoor residual spraying, *ITN* insecticide-treated net

Across the three models of transmission intensity (regional *Pf*PR_2–10_, EA *Pf*PR_2–10_ and MSP zones), intervention coverage did not exceed 60% (Table 2). Additionally, only 2.6% of all enumeration areas had ≥ 95% coverage with at least one intervention in MSP Zone 1 (Fig. 4). These analyses suggest that regional transmission intensity was strongly associated with the likelihood of owning an ITN or having household IRS. MSP zones 1 and 2 were also associated with having an ITN and IRS or either intervention in fully adjusted models (Additional file 1: Table S3).

Given government strategies for targeted distribution of ITNs and IRS, and intra-regional variations in transmission intensity, EA level transmission intensities and MSP zones were assessed to understand whether these explained the distribution of ITNs and IRS in the DHS data better than the regional *Pf*PR_2–10_ model.

A multivariable statistical model with regional *Pf*PR_2–10_ was fitted. Log-likelihood ratio tests were used to assess whether EA *Pf*PR_2–10_ improved the fit of this model, to examine whether EA *Pf*PR_2–10_ better explained the variation in IRS and ITN distribution. All log-likelihood ratio tests found that EA *Pf*PR_2–10_ did not explain the variation in ITN and IRS coverage compared with regional *Pf*PR_2–10_ [p values (1 *df*) ranged from 0.70 to 0.93; Table 6]. This finding highlights that regional malaria transmission indices explain the distribution of ITNs and IRS in these data better than those derived at the EA level, which is consistent with the government MSP intervention strategy. Next, models were fitted using MSP zones for IRS and ITN coverage and compared them with regional *Pf*PR_2–10_. All log-likelihood ratio tests showed that adding MSP zones statistically significantly improved the fit of the model [p values (1 *df*) ranged from < 0.001 to 0.009; Table 6]. These analyses indicate that the Namibian Government’s intervention strategy explains additional variation in the coverage of IRS and ITNs in the 2013 Namibia DHS data.

**Table 6:** Comparison of regional *Pf*PR_2–10_, EA *Pf*PR_2–10_ and MSP zones for predicting the likelihood of having IRS, an ITN or either intervention

Models		IRS			ITN			IRS and/or ITN		
		RR (95% CI)	p-value	LR test p-value	RR (95% CI)	p-value	LR test p-value	RR (95% CI)	p-value	LR test p-value
Model 1	Regional *Pf*PR_2–10_	3.71 (2.29–6.02)	< 0.001		2.20 (1.57–3.10)	< 0.001		2.38 (1.78–3.19)	< 0.001	
Model 2	Regional *Pf*PR_2–10_	3.86 (2.29–6.53)	< 0.001	0.7000	2.19 (1.54–3.13)	< 0.001	0.9347	2.42 (1.79–3.27)	< 0.001	0.7480
	EA *Pf*PR_2–10_	0.96 (0.76–1.20)	0.700		1.01 (0.89–1.13)	0.935		0.98 (0.89–1.08)	0.748	
Model 3	Regional *Pf*PR_2–10_	2.40 (1.41–4.10)	0.001	0.0091	1.43 (1.04–1.97)	0.029	0.0001	1.71 (1.31–2.34)	< 0.001	< 0.0001
	MSP zone	1.69 (1.15–2.50)	0.008		1.69 (1.31–2.18)	< 0.001		1.50 (1.24–1.81)	< 0.001	

Model 1, association between regional *Pf*PR_2–10_ and interventions, adjusted for wealth and residence type, with region and enumeration area added as mixed effects; Model 2, same as Model 1 but additionally adjusted for EA *Pf*PR_2–10_; Model 3, same as Model 1 but additionally adjusted for MSP zones; *Pf*PR_2–10_, *Plasmodium falciparum* parasite rate in those aged 2–10 years; ITN, insecticide-treated net; IRS, indoor residual spraying, LR test, log-likelihood ratio test

p-value corresponds to a log-likelihood ratio test where Models 2 and 3 are respectively nested in Model 1

Given the varying approaches used to assign *Pf*PR_2–10_ values to EAs, further sensitivity analyses were conducted to assess the impact of this on the results. Re-parameterizing EA *Pf*PR_2–10_ metrics did not materially affect the estimates for the coverage of interventions (IRS, ITN, IRS and/or ITN) by EA *Pf*PR_2–10_ category (< 1, 1 to < 5 and ≥ 5%) (Additional file 1: Table S4). Further, these additional EA *Pf*PR_2–10_ models did not improve upon the regional *Pf*PR_2–10_ model for predicting intervention coverage (Additional file 1: Tables S5–S7). Assigning EAs *Pf*PR_2–10_ values of the nearest cell up to 5 km away did not improve on the regional *Pf*PR_2–10_ model for IRS coverage (p = 0.1858), ITN coverage (p = 0.4534) or having either intervention (p = 0.2751) (Additional file 1: Table S5). Additionally, accounting for EA displacement did not improve upon the regional *Pf*PR_2–10_ model for IRS coverage (p = 0.5002), ITN coverage (p = 0.5441) or having either intervention (p = 0.6507) (Additional file 1: Tables S8 and S9).

## Discussion

These detailed analyses indicate that the prevalence of IRS and ITN interventions for malaria in 2013 did not reflect governmental malaria intervention targets in Namibia. In this DHS sample of 9846 households representing 41,314 individuals, and malaria transmission intensity data, the prevalence of at least one intervention (ITN or IRS) was around 34% across Namibia, and 49% in the highest transmission regions in 2013. These analyses highlight the need to include quantitative monitoring of such interventions, to provide a framework to reliably evaluate intervention strategies for malaria.

Operational constraints for IRS delivery have been reported in previous years in Namibia [33]. These constraints included a lack of community acceptability, shortage of human resources, late payments of spray men and challenges in access due to rains and flooding [33]. High levels of community acceptability of ITNs have also historically been difficult to achieve [33]. The need for the government to address operational constraints, particularly human resource capacity to implement these interventions, has been identified [33] and may partly explain the low coverage observed in the DHS.

Low coverage of malaria control interventions is not unique to Namibia; this has also been observed in DHS reports for other southern African countries. The Zambia 2013–2014 DHS found ITN coverage to be 68% and IRS coverage to be 28% [34]. In Zimbabwe, 48% of households in 2015 had an ITN and 21% had IRS [35]. Angola had a lower coverage of interventions than reported by the Namibia DHS, with 7% of households with IRS and 35% that owned an ITN in 2011 [36]. ITN coverage was higher than IRS coverage in all of these countries, consistent with the findings in Namibia.

By contrast to the 2013 Namibia DHS Report and these findings, the Namibia Ministry of Health and Social Services Annual Report 2012/2013 stated that at least 93% of households received these interventions [19]. It was reported that across eight malaria regions, 669,578 households out of a targeted 719,412 structures received IRS by the end of January 2013 [19]. The report also stated that 87,900 ITNs were procured but only 7000 ITNs were distributed at the time of the Annual Report [19]. The DHS identified around 4300 ITNs owned by the survey households collectively, 49% of which were obtained in the previous 12 months but 37% of which were obtained more than 3 years prior to the survey (Additional file 1: Table S10).

The DHS was carried out at least 4 months following the end of the spray season and at the end of the malaria transmission season. As such, the survey was well timed to provide a nationally-representative estimate of the coverage of these interventions by the end of the transmission season.

One of the limitations of this analysis is that it uses data collected as part of a survey investigating a range of health and disease indices, not only malaria. Data were not collected on larviciding practices so it was not possible to include larviciding in vector control estimates. Further, differences in intervention coverage reported by the DHS and the Annual Report could be due to a lack of overlap between households surveyed in the DHS and the households reported to be sprayed in the Annual Report or targeted by the IRS programme. However, the DHS surveyed approximately 2% of all households recorded in the 2011 Population and Housing Census and was designed to be nationally representative. In this context, the DHS appropriately represents the Namibian population based on national census data.

The nature of data collection on vector control methods as part of the DHS may be a further limitation. Information on whether a household received IRS was obtained by asking a household member whether the household had been sprayed against mosquitoes in the last 12 months. It is possible that the household member who answered the question may not have remembered this event or may not have been present at the time of spraying, for example, which would result in under-reporting of IRS. It was also not possible to ascertain which specific households were targeted for spraying by the IRS programme; thus, these estimates may not reflect programme efforts in targeted areas. However, it was observed that, as well as regional transmission intensity, MSP target zones additionally explained the variation in ITN and IRS in the DHS data—suggesting that the DHS data reflect the coverage of government malaria interventions.

Information on ITN ownership and use was gathered from survey questions, in addition to an inventory conducted by the interviewer. The number of ITNs could have been underestimated if not all the ITNs originally distributed were shown to the interviewer, for example, or if the nets were discarded, sold, or used for other purposes, as has been reported elsewhere [37–40]. The number of ITNs may also have been overestimated, as not all ITNs that were reported as part of the DHS were actually observed by the interviewer. The source of the ITNs is also unknown. Whilst these factors together may result in some uncertainty around the estimates of IRS and ITN coverage, they are unlikely to fully account for the difference between estimates provided by the DHS data and governmental reports.

Study inferences may also be limited by the classification of regions and EAs based on MAP data. MAP *Pf*PR_2–10_ values are predicted values and do not necessarily reflect actual levels of transmission intensity in 2013. Using MAP data, Zambezi is classified as being in the 1 to < 5% regional *Pf*PR_2–10_ category; however, other studies and reports have identified this as one of the higher risk regions in Namibia [12, 33, 41]. Re-categorizing Zambezi resulted in only a minor increase in ITN and IRS coverage of no more than 4% for any intervention in the highest transmission regions (Additional file 1: Table S1). *Pf*PR_2–10_ data were also analyzed at both EA and regional levels, with regional level analyses found to better explain variation in ITN and IRS distribution in the DHS data—suggesting that finer-scale geographical data on transmission intensity data does not explain these patterns of ITN and IRS distribution.

## Conclusion

These findings indicate that the prevalence of IRS and ITN interventions for malaria in 2013 did not reflect governmental malaria intervention targets in Namibia. The WHO recommends that “Malaria control and elimination programmes should prioritize delivering either LLINs or IRS at high coverage and to a high standard rather than introducing the second intervention as a means of compensating for deficiencies in the implementation of the first” [42]. Given the relatively low malaria transmission in Namibia and the operational challenges of delivering vector control interventions, it will be relevant to identify the barriers to implement interventions or prioritize the implementation of a single intervention. As countries such as Namibia work towards malaria elimination, high coverage of vector control interventions will be critical, not only to reduce the incidence of malaria but also to prevent resurgence. Such efforts will require quantitative monitoring to assess implementation and provide a framework to reliably evaluate the effectiveness of these interventions and inform future strategies for malaria elimination.

## Additional file

**Additional file 1.** Additional tables.
