# Supplementary material for: A cross-sectional analysis of ITN and IRS coverage in Namibia in 2013
Source: Malar J. 2018 Jul 16;17:264. doi: 10.1186/s12936-018-2417-z (PMC6048889; doi:10.1186/s12936-018-2417-z)
Supplement: Supplementary file 1 — Additional file 1. Additional tables. [file 12936_2018_2417_MOESM1_ESM.docx]

**Additional tables**

| **Table S1:**  **Association of *Pf*PR_2-10_ and wealth quintile with IRS, stratified by residence type** | | | | | | | | |
| --- | --- | --- | --- | --- | --- | --- | --- | --- |
|  | **Urban** | | | | **Rural** | | | |
| **Exposures of interest** | **Model 1** | | **Model 2** | | **Model 1** | | **Model 2** | |
|  | **RR (95% CI)** | **p-value** | **RR (95% CI)** | **p-value** | **RR (95% CI)** | **p-value** | **RR (95% CI)** | **p-value** |
| **Wealth quintile** |  |  |  |  |  |  |  |  |
| Lowest | 1.00 (reference) |  | 1.00 (reference) |  | 1.00 (reference) |  | 1.00 (reference) |  |
| Second | 0.79 (0.27 - 2.28) | 0.660 | 1.23 (0.41 - 3.65) | 0.713 | 0.92 (0.81 - 1.04) | 0.169 | 1.18 (1.03 - 1.34) | 0.018 |
| Middle | 1.21 (0.44 - 3.36) | 0.709 | 1.91 (0.68 - 5.42) | 0.222 | 0.70 (0.61 - 0.81) | <0.001 | 1.16 (0.99 - 1.37) | 0.071 |
| Fourth | 0.91 (0.33 - 2.51) | 0.860 | 1.76 (0.61 - 5.06) | 0.297 | 0.68 (0.57 - 0.81) | <0.001 | 1.25 (1.02 - 1.53) | 0.032 |
| Highest | 0.79 (0.29 - 2.17) | 0.652 | 2.44 (0.83 - 7.18) | 0.105 | 0.48 (0.35 - 0.66) | <0.001 | 1.47 (1.03 - 2.09) | 0.035 |
|  |  |  |  |  |  |  |  |  |
| ***Pf*PR_2-10_** |  |  |  |  |  |  |  |  |
| <1% | 1.00 (reference) |  | 1.00 (reference) |  | 1.00 (reference) |  | 1.00 (reference) |  |
| 1-<5% | 1.23 (0.51 - 2.97) | 0.649 | 1.57 (0.26 - 9.44) | 0.623 | 14.36 (8.39 - 24.58) | <0.001 | 9.86 (2.48 - 39.22) | 0.001 |
| 5-<10% | 12.12 (8.09 - 18.16) | <0.001 | 11.69 (3.47 - 39.36) | <0.001 | 17.85 (10.54 - 30.23) | <0.001 | 22.14 (7.43 - 65.96) | <0.001 |
| Model 1: Univariable \| Model 2: Adjusted for enumeration area and regional clustering and adjusted for other exposures of interest in the table \| IRS: Indoor residual spraying \| *Pf*PR_2-10_: *Plasmodium falciparum* parasite rate in those aged 2 to 10 years | | | | | | | | |

| **Table S2:**  **Association of *Pf*PR_2-10_ and wealth quintile with ITN ownership, stratified by residence type** | | | | | | | | |
| --- | --- | --- | --- | --- | --- | --- | --- | --- |
|  | **Urban** | | | | **Rural** | | | |
| **Exposures of interest** | **Model 1** | | **Model 2** | | **Model 1** | | **Model 2** | |
|  | **RR (95% CI)** | **p-value** | **RR (95% CI)** | **p-value** | **RR (95% CI)** | **p-value** | **RR (95% CI)** | **p-value** |
| **Wealth quintile** |  |  |  |  |  |  |  |  |
| Lowest | 1.00 (reference) |  | 1.00 (reference) |  | 1.00 (reference) |  | 1.00 (reference) |  |
| Second | 1.35 (0.73 - 2.52) | 0.342 | 1.60 (0.85 - 3.00) | 0.141 | 0.98 (0.87 - 1.12) | 0.807 | 1.18 (1.04 - 1.35) | 0.012 |
| Middle | 1.51 (0.82 - 2.77) | 0.186 | 1.86 (1.01 - 3.46) | 0.048 | 0.95 (0.83 - 1.09) | 0.457 | 1.35 (1.17 - 1.57) | <0.001 |
| Fourth | 1.13 (0.62 - 2.07) | 0.686 | 1.78 (0.96 - 3.30) | 0.068 | 0.95 (0.81 - 1.11) | 0.510 | 1.56 (1.31 - 1.86) | <0.001 |
| Highest | 0.94 (0.51 - 1.72) | 0.843 | 1.95 (1.05 - 3.64) | 0.035 | 0.57 (0.42 - 0.77) | <0.001 | 1.29 (0.93 - 1.79) | 0.127 |
|  |  |  |  |  |  |  |  |  |
| ***Pf*PR_2-10_** |  |  |  |  |  |  |  |  |
| <1% | 1.00 (reference) |  | 1.00 (reference) |  | 1.00 (reference) |  | 1.00 (reference) |  |
| 1-<5% | 5.22 (4.15 - 6.57) | <0.001 | 5.63 (2.50 - 12.68) | <0.001 | 5.13 (3.93 - 6.68) | <0.001 | 5.67 (2.62 - 12.26) | <0.001 |
| 5-<10% | 5.48 (4.55 - 6.59) | <0.001 | 6.19 (3.40 - 11.27) | <0.001 | 3.81 (2.96 - 4.92) | <0.001 | 4.62 (2.53 - 8.44) | <0.001 |
| Model 1: Univariable \| Model 2: Adjusted for enumeration area and regional clustering and adjusted for other exposures of interest in the table \| ITN: Insecticide treated net \| *Pf*PR2-10: *Plasmodium falciparum* parasite rate in those aged 2 to 10 years | | | | | | | | |

| **Table S3: Multivariable association between vector control intervention and exposures of interest, adjusted for regional and EA clustering and other covariates, in Namibia 2013 (n=9,597)** | | | | | | | | |
| --- | --- | --- | --- | --- | --- | --- | --- | --- |
| **Exposures of interest** | **IRS** | |  | **ITN*** | |  | **IRS and/or ITN** | |
|  | **RR (95% CI)** | **p-value** |  | **RR (95% CI)** | **p-value** |  | **RR (95% CI)** | **p-value** |
|  |  |  |  |  |  |  |  |  |
| **MSP Zone** |  |  |  |  |  |  |  |  |
| 3 | 1.00 (reference) |  |  | 1.00 (reference) |  |  | 1.00 (reference) |  |
| 2 | 6.99 (2.93 - 16.70) | <0.001 |  | 3.11 (2.10 - 4.61) | <0.001 |  | 3.89 (2.69 - 5.62) | <0.001 |
| 1 | 11.62 (4.89 - 27.60) | <0.001 |  | 5.36 (3.55 - 8.09) | <0.001 |  | 5.62 (3.82 - 8.25) | <0.001 |
|  |  |  |  |  |  |  |  |  |
| **Wealth Quintile** |  |  |  |  |  |  |  |  |
| Lowest | 1.00 (reference) |  |  | 1.00 (reference) |  |  | 1.00 (reference) |  |
| Second | 1.16 (1.02 - 1.33) | 0.026 |  | 1.21 (1.07 - 1.37) | 0.003 |  | 1.12 (1.01 - 1.24) | 0.026 |
| Middle | 1.21 (1.03 - 1.41) | 0.019 |  | 1.40 (1.23 - 1.60) | <0.001 |  | 1.23 (1.10 - 1.38) | <0.001 |
| Fourth | 1.27 (1.05 - 1.53) | 0.015 |  | 1.51 (1.30 - 1.75) | <0.001 |  | 1.31 (1.15 - 1.49) | <0.001 |
| Highest | 1.66 (1.27 - 2.17) | <0.001 |  | 1.52 (1.26 - 1.84) | <0.001 |  | 1.39 (1.18 - 1.63) | <0.001 |
|  |  |  |  |  |  |  |  |  |
| **Residence type** |  |  |  |  |  |  |  |  |
| Urban | 1.00 (reference) |  |  | 1.00 (reference) |  |  | 1.00 (reference) |  |
| Rural | 4.71 (3.59 - 6.17) | <0.001 |  | 1.27 (1.11 - 1.46) | <0.001 |  | 1.57 (1.40 - 1.75) | <0.001 |
|  |  |  |  |  |  |  |  |  |
| *N=9,842 households  MSP: Malaria Strategic Plan \| IRS: Indoor residual spraying \| ITN: Insecticide-treated net \| EA: Enumeration Area | | | | | | | | |

| **Table S4: Table showing the differences in intervention coverage by various models of EA *Pf*PR_2-10_** | | | | | | | | | | | | |
| --- | --- | --- | --- | --- | --- | --- | --- | --- | --- | --- | --- | --- |
|  | **IRS** | | | |  | **ITN** | | |  | **ITN and/or IRS** | | |
|  | **No** | **Yes** | **don't know** | **p-value** |  | **No ITN** | **At least one ITN** | **p-value** |  | **None** | **ITN and/or IRS** | **p-value** |
|  | No. (%) | No. (%) | No. (%) |  |  | No. (%) | No. (%) |  |  | No. (%) | No. (%) |  |
|  |  |  |  |  |  |  |  |  |  |  |  |  |
| **EA *Pf*PR_2-10_ Model A** |  |  |  |  |  |  |  |  |  |  |  |  |
| <1% | 3,942 (95.1) | 110 (2.7) | 95 (2.3) | <0.001 |  | 3,791 (91.4) | 359 (8.7) | <0.001 |  | 3,627 (89.5) | 425 (10.5) | <0.001 |
| 1-<5% | 776 (66.6) | 355 (30.5) | 34 (2.9) |  |  | 607 (51.1) | 558 (47.9) |  |  | 457 (40.4) | 674 (59.6) |  |
| >5% | 3,203 (70.7) | 1,211 (26.7) | 116 (2.6) |  |  | 3,075 (67.9) | 1,456 (32.1) |  |  | 2,286 (51.8) | 2,128 (48.2) |  |
|  |  |  |  |  |  |  |  |  |  |  |  |  |
| **EA *Pf*PR_2-10_ Model B** |  |  |  |  |  |  |  |  |  |  |  |  |
| <1% | 3,810 (94.9) | 109 (2.7) | 95 (2.4) | <0.001 |  | 3,682 (91.7) | 335 (8.3) | <0.001 |  | 3,519 (89.8) | 400 (10.2) | <0.001 |
| 1-<5% | 776 (66.6) | 355 (30.5) | 34 (2.9) |  |  | 607 (52.1) | 558 (47.9) |  |  | 457 (40.4) | 674 (59.6) |  |
| >5% | 3,335 (71.5) | 1,212 (26.0) | 116 (2.5) |  |  | 3,184 (68.3) | 1,480 (31.7) |  |  | 2,394 (52.7) | 2,153 (47.4) |  |
|  |  |  |  |  |  |  |  |  |  |  |  |  |
| **EA *Pf*PR_2-10_ Model C** |  |  |  |  |  |  |  |  |  |  |  |  |
| <1% | 3,612 (95.1) | 95 (2.5) | 90 (2.4) | <0.001 |  | 3,499 (92.1) | 301 (7.9) | <0.001 |  | 3,349 (90.3) | 358 (9.7) | <0.001 |
| 1-<5% | 776 (66.6) | 355 (30.5) | 34 (2.9) |  |  | 607 (52.1) | 558 (47.9) |  |  | 457 (40.4) | 674 (59.6) |  |
| >5% | 3,533 (72.4) | 1,226 (25.1) | 121 (2.5) |  |  | 3,367 (69.0) | 1,514 (31.0) |  |  | 2,564 (53.9) | 2,195 (46.1) |  |
|  |  |  |  |  |  |  |  |  |  |  |  |  |
| **EA *Pf*PR_2-10_ Model D** |  |  |  |  |  |  |  |  |  |  |  |  |
| <1% | 3,541 (96.1) | 56 (1.5) | 88 (2.4) | <0.001 |  | 3,411 (92.5) | 277 (7.5) | <0.001 |  | 3,288 (91.4) | 309 (8.6) | <0.001 |
| 1-<5% | 776 (66.6) | 355 (30.5) | 34 (2.9) |  |  | 607 (52.1) | 558 (47.9) |  |  | 457 (40.4) | 674 (59.6) |  |
| >5% | 3,604 (72.2) | 1,265 (25.3) | 123 (2.5) |  |  | 3,455 (69.2) | 1,538 (30.8) |  |  | 2,625 (53.9) | 2,244 (46.1) |  |
|  |  |  |  |  |  |  |  |  |  |  |  |  |
| *Pf*PR2-10: *Plasmodium falciparum* parasite rate in those aged 2 to 10 years \| IRS: Indoor residual spraying \| ITN: Insecticide-treated net \| EA: enumeration area  Model A: EA *Pf*PR where EAs outside of the *Pf*PR raster boundary were assigned a value of zero  Model B: EA *Pf*PR where EAs at a distance >5 Km from the nearest raster cell were assigned a value of zero  Model C: EA *Pf*PR where EAs at a distance >10 Km from the nearest raster cell were assigned a value of zero  Model D: EA *Pf*PR where EAs at a distance >20 Km from the nearest raster cell were assigned a value of zero | | | | | | | | | | | | |

| **Table S5: Comparison of regional *Pf*PR_2-10_, EA *Pf*PR_2-10_ (Model B*) and MSP Zones for predicting the likelihood of having IRS, an ITN or either intervention.** | | | | | | | | | | | | |
| --- | --- | --- | --- | --- | --- | --- | --- | --- | --- | --- | --- | --- |
| **Models** | | **IRS** | | |  | **ITN** | | |  | **IRS and/or ITN** | | |
|  |  | **RR (95% CI)** | **p-value** | **LR test p-value** |  | **RR (95% CI)** | **p-value** | **LR test p-value** |  | **RR (95% CI)** | **p-value** | **LR test p-value** |
|  |  |  |  |  |  |  |  |  |  |  |  |  |
| **Model 1:** | Regional *Pf*PR_2-10_ | 3.71 (2.29 - 6.02) | <0.001 |  |  | 2.20 (1.57 - 3.10) | <0.001 |  |  | 2.38 (1.78 - 3.19) | <0.001 |  |
|  |  |  |  |  |  |  |  |  |  |  |  |  |
|  |  |  |  |  |  |  |  |  |  |  |  |  |
| **Model 2:** | Regional *Pf*PR_2-10_ | 4.29 (2.53 - 7.28) | <0.001 | 0.1858 |  | 2.30 (1.61 - 3.29) | <0.001 | 0.4534 |  | 2.50 (1.85 - 3.39) | <0.001 | 0.2751 |
|  | EA *Pf*PR_2-10_* | 0.85 (0.66 - 1.08) | 0.183 |  |  | 0.95 (0.84 - 1.08) | 0.452 |  |  | 0.95 (0.86 - 1.05) | 0.273 |  |
|  |  |  |  |  |  |  |  |  |  |  |  |  |
| **Model 3:** | Regional *Pf*PR_2-10_ | 2.40 (1.41 - 4.10) | 0.001 | 0.0091 |  | 1.43 (1.04 - 1.97) | 0.029 | 0.0001 |  | 1.71 (1.31 - 2.34) | <0.001 | <0.0001 |
|  | MSP Zone | 1.69 (1.15 - 2.50) | 0.008 |  |  | 1.69 (1.31 - 2.18) | <0.001 |  |  | 1.50 (1.24 - 1.81) | <0.001 |  |
| *** Model B: EA *Pf*PR_2-10_ model assigns EAs the raster cell value up to 5 Km away  *Pf*PR2-10: *Plasmodium falciparum* parasite rate in those aged 2 to 10 years \| ITN: Insecticide-treated net \| IRS: Indoor residual spraying \| LR test: Log-likelihood ratio test \| EA: enumeration area \| MSP: Malaria Strategic Plan \| p-value corresponds to a log-likelihood ratio test where Model 2 and Model 3 are respectively nested in Model 1. | | | | | | | | | | | | |
| Model 1: Association between regional *Pf*PR_2-10_ and interventions, adjusted for wealth and residence type, with region and enumeration area added as mixed effects | | | | | | | | | | | | |
| Model 2: Same as Model 1 but additionally adjusted for EA *Pf*PR_2-10_ | | | | | | | | | | | | |
| Model 3: Same as Model 1 but additionally adjusted for MSP Zones | | | | | | | | | | | | |

| **Table S6: Comparison of regional *Pf*PR_2-10_, EA *Pf*PR_2-10_ (Model C*) and MSP Zones for predicting the likelihood of having IRS, an ITN or either intervention.** | | | | | | | | | | | | |
| --- | --- | --- | --- | --- | --- | --- | --- | --- | --- | --- | --- | --- |
| **Models** | | **IRS** | | |  | **ITN** | | |  | **IRS and/or ITN** | | |
|  |  | **RR (95% CI)** | **p-value** | **LR test p-value** |  | **RR (95% CI)** | **p-value** | **LR test p-value** |  | **RR (95% CI)** | **p-value** | **LR test p-value** |
|  |  |  |  |  |  |  |  |  |  |  |  |  |
| **Model 1:** | Regional *Pf*PR_2-10_ | 3.71 (2.29 - 6.02) | <0.001 |  |  | 2.20 (1.57 - 3.10) | <0.001 |  |  | 2.38 (1.78 - 3.19) | <0.001 |  |
|  |  |  |  |  |  |  |  |  |  |  |  |  |
|  |  |  |  |  |  |  |  |  |  |  |  |  |
| **Model 2:** | Regional *Pf*PR_2-10_ | 4.26 (2.51 - 7.25) | <0.001 | 0.2369 |  | 2.34 (1.63 - 3.37) | <0.001 | 0.3333 |  | 2.54 (1.87 - 3.44) | <0.001 | 0.2120 |
|  | EA *Pf*PR_2-10_* | 0.85 (0.66 - 1.11) | 0.234 |  |  | 0.94 (0.82 - 1.07) | 0.332 |  |  | 0.93 (0.84 - 1.04) | 0.209 |  |
|  |  |  |  |  |  |  |  |  |  |  |  |  |
| **Model 3:** | Regional *Pf*PR_2-10_ | 2.40 (1.41 - 4.10) | 0.001 | 0.0091 |  | 1.43 (1.04 - 1.97) | 0.029 | 0.0001 |  | 1.71 (1.31 - 2.34) | <0.001 | <0.0001 |
|  | MSP Zone | 1.69 (1.15 - 2.50) | 0.008 |  |  | 1.69 (1.31 - 2.18) | <0.001 |  |  | 1.50 (1.24 - 1.81) | <0.001 |  |
| *** Model C: EA *Pf*PR_2-10_ model assigns EAs the raster cell value up to 10 Km away  *Pf*PR2-10: *Plasmodium falciparum* parasite rate in those aged 2 to 10 years \| ITN: Insecticide-treated net \| IRS: Indoor residual spraying \| LR test: Log-likelihood ratio test \| EA: enumeration area \| MSP: Malaria Strategic Plan \| p-value corresponds to a log-likelihood ratio test where Model 2 and Model 3 are respectively nested in Model 1. | | | | | | | | | | | | |
| Model 1: Association between regional *Pf*PR_2-10_ and interventions, adjusted for wealth and residence type, with region and enumeration area added as mixed effects | | | | | | | | | | | | |
| Model 2: Same as Model 1 but additionally adjusted for EA *Pf*PR_2-10_ | | | | | | | | | | | | |
| Model 3: Same as Model 1 but additionally adjusted for MSP Zones | | | | | | | | | | | | |

| **Table S7: Comparison of regional *Pf*PR_2-10_, EA *Pf*PR_2-10_ (Model D*) and MSP Zones for predicting the likelihood of having IRS, an ITN or either intervention.** | | | | | | | | | | | | |
| --- | --- | --- | --- | --- | --- | --- | --- | --- | --- | --- | --- | --- |
| **Models** | | **IRS** | | |  | **ITN** | | |  | **IRS and/or ITN** | | |
|  |  | **RR (95% CI)** | **p-value** | **LR test p-value** |  | **RR (95% CI)** | **p-value** | **LR test p-value** |  | **RR (95% CI)** | **p-value** | **LR test p-value** |
|  |  |  |  |  |  |  |  |  |  |  |  |  |
| **Model 1:** | Regional *Pf*PR_2-10_ | 3.71 (2.29 - 6.02) | <0.001 |  |  | 2.20 (1.57 - 3.10) | <0.001 |  |  | 2.38 (1.78 - 3.19) | <0.001 |  |
|  |  |  |  |  |  |  |  |  |  |  |  |  |
|  |  |  |  |  |  |  |  |  |  |  |  |  |
| **Model 2:** | Regional *Pf*PR_2-10_ | 3.41 (1.97 - 5.92) | <0.001 | 0.5319 |  | 2.34 (1.62 - 3.38) | <0.001 | 0.3728 |  | 2.43 (1.78 - 3.31) | <0.001 | 0.7514 |
|  | EA *Pf*PR_2-10_* | 1.10 (0.82 - 1.47) | 0.532 |  |  | 0.94 (0.81 - 1.08) | 0.371 |  |  | 0.98 (0.87 - 1.10) | 0.751 |  |
|  |  |  |  |  |  |  |  |  |  |  |  |  |
| **Model 3:** | Regional *Pf*PR_2-10_ | 2.40 (1.41 - 4.10) | 0.001 | 0.0091 |  | 1.43 (1.04 - 1.97) | 0.029 | 0.0001 |  | 1.71 (1.31 - 2.34) | <0.001 | <0.0001 |
|  | MSP Zone | 1.69 (1.15 - 2.50) | 0.008 |  |  | 1.69 (1.31 - 2.18) | <0.001 |  |  | 1.50 (1.24 - 1.81) | <0.001 |  |
| *** Model D: EA *Pf*PR_2-10_ model assigns EAs the raster cell value up to 20 Km away  *Pf*PR2-10: *Plasmodium falciparum* parasite rate in those aged 2 to 10 years \| ITN: Insecticide-treated net \| IRS: Indoor residual spraying \| LR test: Log-likelihood ratio test \| EA: enumeration area \| MSP: Malaria Strategic Plan \| p-value corresponds to a log-likelihood ratio test where Model 2 and Model 3 are respectively nested in Model 1. | | | | | | | | | | | | |
| Model 1: Association between regional *Pf*PR_2-10_ and interventions, adjusted for wealth and residence type, with region and enumeration area added as mixed effects | | | | | | | | | | | | |
| Model 2: Same as Model 1 but additionally adjusted for EA *Pf*PR_2-10_ | | | | | | | | | | | | |
| Model 3: Same as Model 1 but additionally adjusted for MSP Zones | | | | | | | | | | | | |

| **Table S8: Comparison of regional *Pf*PR_2-10_, EA *Pf*PR_2-10_ and MSP Zones for predicting the likelihood of having IRS, an ITN or either intervention** | | | | | | | | | | | | |
| --- | --- | --- | --- | --- | --- | --- | --- | --- | --- | --- | --- | --- |
| **Models** | | **IRS** | | |  | **ITN** | | |  | **IRS and/or ITN** | | |
|  |  | **RR (95% CI)** | **p-value** | **LR test p-value** |  | **RR (95% CI)** | **p-value** | **LR test p-value** |  | **RR (95% CI)** | **p-value** | **LR test p-value** |
|  |  |  |  |  |  |  |  |  |  |  |  |  |
| **Model 1:** | Regional *Pf*PR_2-10_ | 3.81 (2.33 – 6.22) | <0.001 |  |  | 2.17 (1.54 – 3.07) | <0.001 |  |  | 2.41 (1.80 - 3.24) | <0.001 |  |
|  |  |  |  |  |  |  |  |  |  |  |  |  |
|  |  |  |  |  |  |  |  |  |  |  |  |  |
| **Model 2:** | Regional *Pf*PR_2-10_ | 4.24 (2.50 – 7.18) | <0.001 | 0.2696 |  | 2.25 (1.06 – 1.36) | <0.001 | 0.5441 |  | 2.53 (1.86 – 3.45) | <0.001 | 0.2659 |
|  | EA *Pf*PR_2-10_ | 0.88 (0.70 – 1.10) | 0.267 |  |  | 0.96 (0.84 – 1.10) | 0.544 |  |  | 0.94 (0.95 – 1.05) | 0.264 |  |
|  |  |  |  |  |  |  |  |  |  |  |  |  |
| **Model 3:** | Regional *Pf*PR_2-10_ | 2.94 (1.65 – 5.22) | 0.001 | 0.1638 |  | 1.25 (0.88 – 1.77) | 0.207 | 0.0001 |  | 1.73 (1.29 – 2.32) | <0.001 | 0.0006 |
|  | MSP Zone | 1.37 (0.88 – 2.12) | 0.159 |  |  | 1.93 (1.41 – 2.64) | <0.001 |  |  | 1.50 (1.19 – 1.89) | 0.001 |  |
| These analyses include a subset of households in EAs for which a mean *Pf*PR_2-10_ value was obtained (IRS N=8,511; ITN N=8,727; ITN and/or IRS N=8,511 households)  *Pf*PR2-10: *Plasmodium falciparum* parasite rate in those aged 2 to 10 years \| ITN: Insecticide-treated net \| IRS: Indoor residual spraying \| LR test: Log-likelihood ratio test \| EA: Enumeration area \| MSP: Malaria Strategic Plan \| p-value corresponds to a log-likelihood ratio test where Model 2 and Model 3 are respectively nested in Model 1.  Model 1: Association between regional *Pf*PR_2-10_ and interventions, adjusted for wealth and residence type, with region and enumeration area added as mixed effects  Model 2: Same as Model 1 but additionally adjusted for EA *Pf*PR_2-10_  Model 3: Same as Model 1 but additionally adjusted for MSP Zones | | | | | | | | | | | | |

| **Table S9: Comparison of regional *Pf*PR_2-10_, mean EA *Pf*PR_2-10_ and MSP Zones for predicting the likelihood of having IRS, an ITN or either intervention** | | | | | | | | | | | | | |
| --- | --- | --- | --- | --- | --- | --- | --- | --- | --- | --- | --- | --- | --- |
| **Models** | | **IRS** | | |  | **ITN** | | |  | **IRS and/or ITN** | | |  |
|  |  | **RR (95% CI)** | **p-value** | **LR test p-value** |  | **RR (95% CI)** | **p-value** | **LR test p-value** |  | **RR (95% CI)** | **p-value** | **LR test p-value** |  |
|  |  |  |  |  |  |  |  |  |  |  |  |  |  |
| **Model 1:** | Regional *Pf*PR_2-10_ | 3.81 (2.33 – 6.22) | <0.001 |  |  | 2.17 (1.54 – 3.07) | <0.001 |  |  | 2.41 (1.80 - 3.24) | <0.001 |  |  |
|  |  |  |  |  |  |  |  |  |  |  |  |  |  |
|  |  |  |  |  |  |  |  |  |  |  |  |  |  |
| **Model 2:** | Regional *Pf*PR_2-10_ | 4.05 (2.40 – 6.82) | <0.001 | 0.5002 |  | 2.18 (1.52 – 3.12) | <0.001 | 0.5441 |  | 2.46 (1.81 – 3.34) | <0.001 | 0.6507 |  |
|  | Mean EA *Pf*PR_2-10_ | 0.93 (0.74 – 1.16) | 0.499 |  |  | 1.00 (0.87 – 1.14) | 0.953 |  |  | 0.98 (0.88 – 1.08) | 0.650 |  |  |
|  |  |  |  |  |  |  |  |  |  |  |  |  |  |
| **Model 3:** | Regional *Pf*PR_2-10_ | 2.94 (1.65 – 5.22) | 0.001 | 0.1638 |  | 1.25 (0.88 – 1.77) | 0.207 | 0.0001 |  | 1.73 (1.29 – 2.32) | <0.001 | 0.0006 |  |
|  | MSP Zone | 1.37 (0.88 – 2.12) | 0.159 |  |  | 1.93 (1.41 – 2.64) | <0.001 |  |  | 1.50 (1.19 – 1.89) | 0.001 |  |  |
| These analyses include a subset of households in EAs for which a mean *Pf*PR_2-10_ value was obtained (IRS N=8,511; ITN N=8,727; ITN and/or IRS N=8,511 households)  *Pf*PR2-10: *Plasmodium falciparum* parasite rate in those aged 2 to 10 years \| ITN: Insecticide-treated net \| IRS: Indoor residual spraying \| LR test: Log-likelihood ratio test \| EA: enumeration area \| MSP: Malaria Strategic Plan \| p-value corresponds to a log-likelihood ratio test where Model 2 and Model 3 are respectively nested in Model 1.  Model 1: Association between regional *Pf*PR_2-10_ and interventions, adjusted for wealth and residence type, with region and enumeration area added as mixed effects  Model 2: Same as Model 1 but additionally adjusted for EA *Pf*PR_2-10_  Model 3: Same as Model 1 but additionally adjusted for MSP Zones | | | | | | | | | | | | | |

| **Table S10: ITNs identified in the 2013 Namibia DHS and the time obtained** | |
| --- | --- |
| **ITN indicator** | **N (%)** |
| **Total ITNs observed** | 4372 |
| **Nets obtained ≤1 year previous** | 2011 (49.0) |
| **Nets obtained ≤2 years previous** | 474 (11.6) |
| **Nets obtained ≤3 years previous** | 108 (2.6) |
| **Nets obtained >3 years previous** | 1,507 (36.8) |
| **Total ITNs with date obtained** | 4100 (100.0) |
| ITN: insecticide-treated net | |

| **Table S11: Intervention coverage by *Pf*PR_2-10_ categories where Zambezi is allocated to the 5-<10% category** | | | | |
| --- | --- | --- | --- | --- |
| **Re-categorized *Pf*PR*** | **Intervention coverage No. (%)** | | | |
|  | **At least one ITN** | **IRS in previous 12 months** | **ITN and IRS** | **ITN and/or IRS** |
| <1% | 213 (6.1) | 41 (1.2) | 8 (0.2) | 241 (7.1) |
| 1-<5% | 150 (20.9) | 21 (2.9) | 7 (1.0) | 163 (23.0) |
| 5-<10% | 2,010 (35.5) | 1,614 (28.5) | 759 (13.8) | 2,823 (51.3) |
| **Pf*PR_2-10_ re-categorized to include Zambezi in the *Pf*PR_2-10_ 5-<10% category  *Pf*PR_2-10_:  *Plasmodium falciparum* parasite rate in those aged 2 to 10 years \| ITN: insecticide-treated net \| IRS: indoor residual spraying | | | | |
